# CCL2 Responses to *Mycobacterium tuberculosis* Are Associated with Disease Severity in Tuberculosis

**DOI:** 10.1371/journal.pone.0008459

**Published:** 2009-12-29

**Authors:** Zahra Hasan, Jacqueline M. Cliff, Hazel M. Dockrell, Bushra Jamil, Muhammad Irfan, Mussarat Ashraf, Rabia Hussain

**Affiliations:** 1 The Aga Khan University, Karachi, Pakistan; 2 London School of Hygiene and Tropical Medicine, London, United Kingdom; Statens Serum Institute, Denmark

## Abstract

**Background:**

Leucocyte activating chemokines such as CCL2, CCL3, and CXCL8 together with proinflammatory IFNγ, TNFα and downmodulatory IL10 play a central role in the restriction of *M. tuberculosis* infections, but is unclear whether these markers are indicative of tuberculosis disease severity.

**Methodology:**

We investigated live *M. tuberculosis*- and *M. bovis* BCG- induced peripheral blood mononuclear cell responses in patients with tuberculosis (TB) and healthy endemic controls (ECs, n = 36). TB patients comprised pulmonary (PTB, n = 34) and extrapulmonary groups, subdivided into those with less severe localized extrapulmonary TB (L-ETB, n = 16) or severe disseminated ETB (D-ETB, n = 16). Secretion of CCL2, IFNγ, IL10 and CCL3, and mRNA expression of CCL2, TNFα, CCL3 and CXCL8 were determined.

**Results:**

*M. tuberculosis*- and BCG- induced CCL2 secretion was significantly increased in both PTB and D-ETB (p<0.05, p<0.01) as compared with L-ETB patients. CCL2 secretion in response to *M. tuberculosis* was significantly greater than to BCG in the PTB and D-ETB groups. *M. tuberculosis-*induced CCL2 mRNA transcription was greater in PTB than L-ETB (p = 0.023), while CCL2 was reduced in L-ETB as compared with D-ETB (p = 0.005) patients. *M. tuberculosis* –induced IFNγ was greater in L-ETB than PTB (p = 0.04), while BCG-induced IFNγ was greater in L-ETB as compared with D-ETB patients (p = 0.036). TNFα mRNA expression was raised in PTB as compared with L-ETB group in response to *M. tuberculosis* (p = 0.02) and BCG (p = 0.03). *Mycobacterium*-induced CCL3 and CXCL8 was comparable between TB groups.

**Conclusions:**

The increased CCL2 and TNFα in PTB patients may support effective leucocyte recruitment and *M. tuberculosis* localization. CCL2 alone is associated with severity of TB, possibly due to increased systemic inflammation found in severe disseminated TB or due to increased monocyte infiltration to lung parenchyma in pulmonary disease.

## Introduction

Tuberculosis (TB) causes 1.8 million deaths annually with 9.27 million incident cases of which the majority (55%) are in Asia [1]. Although the primary disease remains at pulmonary sites, extrapulmonary disease is common especially in high TB burden settings [2], [3] or where there is a high rate of human immunodeficiency virus (HIV) co-prevalence [4].

Protective immunity against *Mycobacterium tuberculosis* is dependent on the interplay between activated T cells, macrophages and other leucocytes. Proinflammatory cytokines such as, interferon gamma (IFN)-γ, tumor necrosis factor-alpha (TNF)-α, interleukin (IL)-12 are essential for protective immunity against *M. tuberculosis* [5], [6]. IL-10 produced by macrophages is important in regulating the TH1 cytokine balance and down regulates proinflammatory responses [7].

Small molecular weight (8–10 kDa) chemotactic cytokines or, chemokines are responsible for regulating the migration, trafficking, homing and activation of monocytes, macrophages and other leucocytes. An effective granulomatous response is essential for the restriction of *M. tuberculosis* infection. TNFα which is essential for macrophage activation and granuloma formation [8], [9] also influences the expression of chemokines by macrophages and mediates effective recruitment of leucocytes via the CC chemokines; CCL2 (monocyte chemoattractant protein (MCP)-1), CCL3 (macrophage inflammatory protein (MIP)- 1α), CCL4 (macrophage inflammatory protein (MIP)- 1β), CCL5 (regulated on activation normal T cell expressed and secreted: RANTES) and CXC chemokines; CXCL8 (IL8), CXCL9 (monokine induced by IFNγ: MIG) and CXCL10 (IFNγ inducible 10kD protein: IP10) [10]–[13].

CCL2 and CCL3 are primarily secreted by monocytes, macrophages and dendritic cells. Responsiveness to CCL2 is dependent on its receptor CCR2, and CCL2 is a potent activator of cells which express CCR2 such as, monocytes, macrophages, CD4+ T cells and immature dendritic cells [14]. CCL2 is essential for granuloma formation [15] and plays a critical role in protection against tuberculosis in the murine model [16]. Chemokines CCL3, CCL4 and CCL5 function together with IFNγ as type 1 proinflammatory chemokines [17]. *M. tuberculosis* infection of macrophages results in the induction of CCL3, CCL4 and CCL5 and these are required for inhibition of its growth [18]. CXC chemokines are predominantly secreted by polymorphonuclear cells and CXCL8 is the most potent chemotactic agent for neutrophils and T lymphocytes [19], [20]. It plays a role in the recruitment of lymphocytes and monocyte to pleural space in TB patients [21], as a result of CXCL8 production by macrophages and mesothelial cells [22].

It remains a challenge to try to identify molecular markers which may be indicative of tuberculosis infection in the host. Most TB studies have focused on patients with pulmonary tuberculosis (PTB). However, it has been shown that the magnitude and regulation of IFNγ, CCL2 and CXCL9 may differ between the host responses of patient with PTB or extrapulmonary TB (ETB) [23]–[25]. In addition, within extrapulmonary TB, the relationship between IFNγ and IL10 regulates the outcome of infection and affects the severity of disease [26]. TNFα gene expression has been shown to be increased in patients with extrapulmonary TB [27]. CXCL8 levels are raised in the sera of patients with TB patients and have been shown to be associated with unfavorable outcome of the disease [28]. Most work on transcriptional profiles of *M. tuberculosis* infected cells have been performed in the murine model [29], [30] or immortalized cells [31], with some recent work in patients with tuberculous meningitis [32]. As these cytokines and chemokines had previously been previously been shown to be differentially secreted according to disease site (pulmonary and extrapulmonary) and also disease severity, we chose to study CCL2, IFNγ, IL10, CCL3, TNFα and CXCL8 in response to *Mycobacterium* infection of peripheral blood mononuclear cells (PBMCs).

BCG vaccination coverage in the Pakistani population is approximately 70% [33] TB but transmission rates remain high with an incidence of 181/100,000 population [1]. Responses to virulent *M. tuberculosis* can differ from those of attenuated avirulent organisms such as, *M. bovis* BCG [34]. We have employed both live virulent *M. tuberculosis* and non-pathogenic *M. bovis* BCG in this study in order to assess whether it was possible to differentiate between immune responses to virulent and avirulent mycobacteria against a background of high transmission, in addition to environmental exposure to cross mycobacteria and wide BCG coverage. BCG vaccinated healthy controls (ECs) can be both tuberculin test positive (TST+) and negative (TST-). It has been shown recently that in clinically health individuals *Mycobacterium*-specific immune responses differ between those with tuberculin positive and negative reactions [35]. Therefore, we have separately described responses of TST+ and TST- ECs and compared them with those of patients with tuberculosis. We have investigated chemokine and cytokine responses in tuberculosis patients with differing clinical severity and sites including PTB and ETB, with a view to identifying markers of clinical disease severity.

## Materials and Methods

### Ethics Statement

This work received approval from the Ethical Review Committee, The Aga Khan University, Karachi, Pakistan.

### Subject Selection

TB patients in this study are a subset of a larger study and have been described previously [36]. Patients were recruited from the out-patient clinics of the Aga Khan University Hospital and Medical College (AKUH) and Masoomeen Hospital, Karachi. The subjects were all unrelated. All study subjects were examined, evaluated and recruited by infectious diseases consultants. The patients were newly diagnosed with ≤7 days of anti-tuberculous therapy (ATT). All samples were taken with written informed consent from participants. Patients had no significant co-morbid conditions including diabetes mellitus, chronic renal failure, and chronic liver disease and were also not on any corticosteroid therapy. Although Pakistan is a low HIV prevalence setting, all patients were screened and found to be HIV negative. Patients with pulmonary TB (PTB, n = 34) were diagnosed by clinical examination, chest X-ray, sputum acid fast bacillus (AFB) Ziehl Neelsen straining, AFB culture and /or clinical response to treatment (as assessed by resolution of fever, cough and weight gain). Patients were diagnosed as having either minimal or moderately advanced disease based on the extent of lung tissue involvement [37], [38]. Of the PTB patients, 9 had minimal, while 25 had moderately advanced disease.

Patients with extrapulmonary TB (ETB) were stratified into disease severity groups according to the WHO ranking of clinical disease severity based on extent of disease and anatomical site and number of distal sites involved [39]. TB of the lymph nodes, unilateral pleural effusion, bone (excluding spine), peripheral join and skin was classified as less severe. TB of the meninges, pericardium, peritoneal cavity, bilateral or extensive pleural effusion, spine, intestines, or miliary TB was classified as severe. Sixteen patients were placed in the category of less severe localized ETB (L-ETB), comprising tuberculous lymphadenopathy. All L-ETB patients were confirmed on histological findings consistent with tuberculosis. Sixteen patients were classified as severe disseminated ETB (D-ETB). Diagnostic criteria used for D-ETB are provided in Table 1. Diagnosis of meningeal TB was based on CSF biochemical findings, supported by AFB culture and findings on contrast-enhanced CT scan and/or MRI. Pleural TB was diagnosed on the basis of pleural fluid biochemical findings, AFB culture, histopathological findings on pleural biopsy and supportive radiological evidence on X-rays and/or contrast-enhanced CT scan.

**Table 1:** Diagnostic criteria for patients with severe disseminated extrapulmonary tuberculosis (D-ETB).

No.	Disease Site	Abscess	Microscopy^a^	Radiology^b^	AFBC^c^	Histopathology^d^
1	Spine	Yes		Yes		Positive
2	Spine	Yes		Yes	Negative	Positive
3	Spine	Yes		Yes		
4	Spine	Yes		Yes		
5	Spine	Yes		Yes		
6	Spine	Yes		Yes		
7	Spine	Yes		Yes		
8	Spine		Negative	Yes	Positive	Positive
9	Intestinal			Yes		Positive
10	Meninges^e^			Yes	Negative	
11	Meninges^e^			Yes	Negative	
12	Meninges		Positive	Yes	Positive	Positive
13	Meninges		Positive	Yes	Positive	
14	Meninges^e^		Negative	Yes	Negative	
15	Meninges^e^					Positive
16	Miliary			Yes		

a indicates acid fast bacilli staining of smears.

b includes Xray, MRI or CT imaging characteristic of tuberculosis.

c acid fast bacilli culture using BACTEC radiometric assay, Becton Dickinson, USA.

d biopsy results indicate caseating or necrotic granulomatous inflammation indicative of *M. tuberculosis* infection.

e showed a favorable clinical response to anti-tuberculous treatment.

BCG-vaccinated asymptomatic healthy volunteers who were staff at AKU with no known exposure to TB were used as endemic controls (ECs). BCG vaccination was assessed based on the presence of a BCG scar. No member of the control group had a household member with tuberculosis nor did they have any relationship to any of the patients recruited in the study. All volunteers had a normal chest X-Ray. Tuberculin skin testing (TST) was assessed by intradermal administration of five tuberculin units on the volar surface of the right arm subcutaneously, and read by a single reader at 48 h. An induration of ≥10mm was used as a cutoff for positive responses. Both TST- (n = 19) and TST+ (n = 17) ECs were included in the study.

### *Mycobacterium* Culture

*M. tuberculosis* (H37Rv) was acquired from ATCC and used as described previously [40]. The *M. bovis* BCG Montreal vaccine strain was used as the non-pathogenic strain. All strains were grown to logarithmic phase in 7H9 Middlebrook medium supplemented with 0.02% glycerol, 10% albumin dextrose catalase (ADC) Middlebrook enrichment and 0.5% Tween-80 (all from Difco Laboratories, Detroit, MI, USA). Aliquots of mycobacteria were frozen in growth medium containing 15% glycerol and stored at -70°C. For the infection assay, aliquots of mycobacteria were freshly thawed, washed three times in PBS and diluted as required for the infection. To avoid mycobacterial clumping, the cell suspension was sonicated briefly then allowed to stand for 5 min to allow large clumps to settle, leaving behind a single cell suspension [40]. A mycobacterial innoculum was also plated out for each assay to determine bacterial viability which was greater than 80% in each case.

### Infection of Peripheral Blood Mononuclear Cells with *Mycobacteria*

Peripheral blood mononuclear cells (PBMCs) were obtained by gradient separation of whole blood using Histopaque (GIBCO-BRL, USA). Cells were counted using a hemacytometer and plated at 10^6^ per well in a 24 well tissue culture plate in 1 ml. *M. tuberculosis* and BCG inoculation of 10^6^ CFU/ml (infection ratio of 1) was added to each well containing PBMCs. The time course and dose response to *Mycobacterium* infection of PBMCs has been described previously [41]. All supernatants were collected at 18 h post-stimulation for cytokine and chemokine measurements. Samples were centrifuged to collect any cellular debris, aliquoted and stored at −70 C until tested. Cell monolayers were harvested directly in Trizol reagent (Invitrogen, USA) for extraction of total RNA and stored at −70 C. *M. tuberculosis*-induced responses were tested in all 66 TB patients recruited in this study. However, 63/66 were used for BCG-stimulation experiments due to a lower yield of PBMCs from these 3 patients.

### ELISA for IFNγ, IL-10, CCL2 and CCL3

IFNγ and IL-10 were detected in supernatants of stimulated PBMCs by using standards and ELISA reagents obtained from Endogen (Rockford, IL, USA). Cytokines were measured using a sandwich ELISA technique according to the manufacturer's instructions and as reported previously [23]. Recombinant human cytokine was used to obtain a dose response curve with a range of detection from 3.9–1000 pg/ml. All experimental samples were tested in duplicate. CCL2 and CCL3 standards and monoclonal antibody pairs for capture and detection were obtained from R&D Systems (Abingdon, UK). All measurements were carried out according to the manufacturer's recommendations and as described previously [23]. Recombinant human chemokine was used to obtain a dose response curve with a range of detection from 6.25–500 pg/ml for CCL3, and 6.25–1000 pg/ml for CCL2.

### Real Time Quantitative RT-PCR for Cytokine Gene Expression Quantification

RNA was extracted from PBMC samples stored in Trizol reagent as per the manufacturer's instructions. RNA was heated at 70°C to denature and quantified using the NanoDrop ND1000 (NanoDrop Technologies, USA). Total RNA (1 µg) was reverse transcribed (RT) using MuLV reverse transcriptase (Invitrogen, USA) in a volume of 20 ul and cDNA was further diluted to 25 ul and used in PCR reactions.

The absolute quantification method was used to determined gene expression in cells. Individual standards were prepared from gene specific PCR products generated using a conventional PCR machine, electrophoresed on an agarose gel and subsequently extracted and quantified. Quantification of cDNA product was carried out using a fluorescent quantification assay Quant-IT DNA Assay (Molecular Probes, USA). dsDNA concentration was calculated as copies/ul using the formula: *Copies/ul  =  Xg/ul DNA ÷ (product size in bpx660)×6.022×10 ^23^*.

Standard curves were used 10^6^–10^1^ copies/ well of each gene. For each sample, gene expression PCR was carried out using 2 µl of cDNA template with sequence specific primers. PCR was performed for the human acidic ribosomal protein (HuPO) house keeping gene [42]. Primers for CCL2 (F- CCCCAGTCACCTGCTGTTAT, R- AGATCTCCTTGGCCACAATG) and CCL3 (F- TGCTGCTTCAGCTACACCTC, R- TTTCTGGACCCACTCCTCAC) were from RT primer DB (http://rtprimerdb.org). CXCL8 (F- GCTCTGTGTGAAGGTGCAG, R- TCTGCACCCAGTTTTCCTTG) and TNFα (F- TGCTTGTTCCTCAGCCTCTT, R- GGTTTGCTACAACATGGCTAC) sequences were by courtesy of Martin Holland, LSHTM, UK. All assays employed incorporation of the SYBR Green dye (BIORAD laboratories, USA). Cytokine gene expression ratios were calculated in each case after normalization against HuPO (F- GCTTCCTGGAGGGTGTCC, R- GGACTCGTTTGTACCCGTTG). Typical assay conditions employed were: initial denaturation 50 C, 2 min; 95 C, 15 min; 40 cycles 95C, 15s, 60 C,60 s. This was followed by a melting curve dissociation analysis to check specificity of PCR products. All experiments were carried out using an iCycler real-time PCR machine, BIORAD Laboratories, USA. Data are depicted as fold increase in each target gene per 100 copies. All genes were normalized to the human acidic ribosomal protein (HuP0) housekeeping gene [43]. Fold increase in gene expression were determined based on results of stimulated cells as a fold change in gene expression as compared with basal levels in unstimulated cells. Of the total 66 TB patients recruited in this study, gene expression studies were performed on 50 TB patients. Of these, PBMC gene expression data was available on 50 donors stimulated with *M. tuberculosis* and 44 TB patient PBMCs stimulated with BCG.

### Statistical Analysis

All data were analyzed using the Statistical Package for Social Sciences software (SPSS). Analysis of non-parametric data was performed using the Mann-Whitney U test and the Kruskal-Wallis test as was appropriate. P values≤0.05 were considered to indicate significant differences between groups.

## Results

The hematological characteristics of patients and controls included in the study are provided in Table 2, with each group shown separately. While BCG vaccination is administered at birth as part of the National Expanded Immunization Program in the country its coverage is at best up to 70% [33]. Therefore, we also documented the presence of a BCG scar in subjects to determine whether they had a history of BCG vaccination. TB transmission rates in the country are high with an incidence of 181/100,000 therefore only the TST- EC group can be considered as un-infected by *M. tuberculosis*. The TST+ EC group is also clinically healthy but it not possible to assess whether their positive tuberculin reaction is attributable to BCG vaccination or exposure to environmental mycobacteria or even *M. tuberculosis*. Hence we have considered TST+ and TST- ECs separately.

**Table 2:** Characteristics of tuberculosis patients and controls in the study.

Group	TST- ECs	TST+ ECs	PTB	L-ETB	D-ETB	p-value
	Median (IQR)	Median (IQR)	Median (IQR)	Median (IQR)	Median (IQR^a^)	
**N**	19	17	34	16	16	
**Age (y)**	25 (5)	26 (12.5)	24.5 (12)	**30 (20)**	**31.5 (40.5)**	0.034*
**Male : Female**	10 vs 9	6 vs 11	13 vs 21	7 vs 9	8 vs 8	
**BCG vaccinees ^#^ (%)**	100	100	41.2	**81.3**	37.5	0.029 *
**Hb (g/dL)**	13.6 (1.9)	12.8 (2.5)	11.8 (2.4)	11.9 (3)	11.9 (2.8)	*0.085*
**TLC (10e^9^/L)**	7.5 (1.9)	7.5 (1.9)	8.1 (4.6)	7.1 (2.5)	7.4 (4.1)	*0.403*
**Lymphocytes (10e^9^/L)**	2.3 (0.7)	2.3 (1.1)	**5.7 (4.6)**	**3.7 (1.7)**	**5.2 (3.4)**	<0.001*
**Monocytes (10e^8^/L)**	4.9 (2.3)	4.9 (2)	**13.6 (8.2)**	**18.6 (12.8)**	**13.8 (7.1)**	*<0.001*
**Neutrophils (10e^9^/L)**	**4.4 (1.2)**	**3.8 (1)**	0.5 (0.5)	0.6 (0.3)	0.5 (0.2)	<0.001*

TST, tuberculin skin test; TST+ individuals had an induration ≥10 mm in size.

PTB, pulmonary TB; localized extrapulmonary TB, L-ETB; disseminated ETB, D-ETB.

IQR, interquartile range between 25th and 75th percentile.

‘#’ based on presence of BCG scar.

‘*’ denotes p<0.05 using the Kruskal-Wallis nonparametric test, values in bold indicate those which are significantly higher.

We found that there was a significant difference between the age groups of patients with TB and healthy controls, (p = 0.034). Patients with both localized ETB and disseminated ETB were older than PTB patients (p = 0.011, p = 0.042, respectively, using Mann-Whitney U nonparametric analysis). As indicated, there was a significant difference between BCG vaccinees in TB patient groups, (p = 0.029). Patients with L-ETB had greater numbers of BCG vaccinees than those with either PTB or D-ETB. There was no relationship between the age of patients and their BCG scar status.

Data indicated an increase in lymphocyte, monocyte and neutrophil counts in TB patients when compared with endemic controls, and is in agreement with previous studies [44].

### Increased *M. tuberculosis* – and BCG- Induced CCL2 Secretion in Patients with TB

It is important to understand the difference between *M. bovis* BCG vaccination induced immunity and that elicited in response to challenge by either *M. tuberculosis* or *M. bovis* BCG. In order to establish the specificity of responses to *Mycobacteria*, we first determined *M. tuberculosis-* and *M. bovis* BCG- induced chemokine and cytokine responses in healthy endemic controls (ECs; TST- (N = 19), TST+ (N = 17) as compared with those of patients with tuberculosis. As shown in Table 3, spontaneous secretion of CCL2 from unstimulated PBMCs of controls and TB patients was comparable. *M. tuberculosis* –induced CCL2 was significantly greater in PBMCs of TB patients as compared with both TST- and TST+ ECs (p<0.001). CCL2 responses to *M. tuberculosis* and BCG showed a parallel trend although the magnitude of secretion from TB patients in response to *M. tuberculosis* was significantly greater as compared with BCG (p<0.001).

**Table 3:** Increased *M. tuberculosis*- and *M. bovis* BCG - induced CCL2 and IL10 and decreased IFNγ responses in TB patients.

**Unstimulated cells**				
Group (n)	CCL2	IFNγ	IL10	CCL3
	Median (IQR)	Median (IQR)	Median (IQR)	Median (IQR)
TST- ECs (n = 19)	0 (173)	11 (86)	0 (2)	197 (1384)
TST+ ECs (n = 17)	832 (1266)	12 (39)	6.1 (39)	618 (2458)
TB (n = 66)	0 (415)	5.1 (33)	6 (42)	211 (996)
*p value*	*0.147*	*0.491*	*0.051*	*0.195*
***M. tuberculosis*** **-induced responses**				
Group (n)	δ CCL2	δ IFNγ	δ IL10	δ CCL3
	Median (IQR)	Median (IQR)	Median (IQR)	Median (IQR)
TST- ECs (n = 19)	0 (0)	798 (1446)	0 (47)	1827 (1679)
TST+ ECs (n = 17)	0 (0)	**1740 (2564)**	0 (0)	1441 (2342)
TB (n = 66)	**1234 (4993)**	389 (945)	**217 (549)**	1114 (1677)
*p value*	<0.001 *	0.042 *	<0.001 *	*0.84*
**BCG-induced responses**				
Group (n)	δ CCL2	δ IFNγ	δ IL10	δ CCL3
	Median (IQR)	Median (IQR)	Median (IQR)	Median (IQR)
TST- ECs (n = 19)	0 (0)	**707 (786)**	0 (12)	1992 (1420)
TST+ ECs (n = 17)	0 (0)	**1279 (1824)**	0 (0.8)	763 (2348)
TB (n = 63)	**204 (798)**	405 (669)	**92 (221)**	1194 (1487)
*p value*	<0.001 *	<0.01 *	<0.001 *	*0.363*

ECs, healthy endemic controls; TST, tuberculin skin test; TB, patients with tuberculosis.

‘δ’ denotes cytokine secretion after background subtraction in each case.

‘*’ denotes p<0.05 using the Kruskal-Wallis nonparametric test; values in bold indicate those which are significantly higher.

IQR, interquartile range between 25^th^ and 75^th^ percentile.

### Decreased *M. tuberculosis* – and BCG- Induced IFNγ and Increased IL10 Secretion in Patients with TB

IFNγ in coordination with IL10 plays a key role in protective immunity against *M. tuberculosis* by regulating effector responses in tuberculosis. *M. tuberculosis*-induced IFNγ levels were significantly lower in TB patients (p = 0.042, Kruskal Wallis analysis; Table 3). *M. tuberculosis*-induced IFNγ did not differ significantly between the TST stratified EC groups. However, responses of TB patients were significantly lower than TST+ ECs (p = 0.022, Mann-Whitney U test), and although there was trend that *M. tuberculosis*-induced IFNγ was also lower in patients than TST- ECs, this did not reach significance.

BCG-induced IFNγ secretion was significantly lower in TB patients as compared with ECs (p<0.01, Kruskal Wallis analysis). TB patients showed lower IFNγ responses compared to both TST- and TST+ ECs (p<0.001, p<0.001, respectively, Mann Whitney U test). This suggests that although the trend of *M. tuberculosis*- and BCG-induced IFNγ secretion was similar in TB patients, the responses differed in TST+ and TST- ECs control groups indicating that the immune responses measured here were specific to the mycobacterial stimulus. Also, increased IFNγ responses in the TST+ EC group may reflect previous exposure leading to increased IFNγ responses due to activation of effector T memory cells in this group.

*M. tuberculosis*-induced IL10 levels were significantly raised in TB patients as compared with both TST- and TST+ ECs (p<0.001, Kruskal Wallis test). Similarly, BCG-induced IL10 was also greater in TB patients as compared with both TST- and TST+ ECs (p<0.001, Kruskal Wallis test).

### *Mycobacterium tuberculosis* – and BCG- Induced CCL3 Secretion in Patients with TB

CCL3 has been shown to be inhibitory for *M tuberculosis* growth in macrophages [45] and has been shown to be upregulated in *M. tuberculosis* infected macrophages [46]. We determined *Mycobacterium-* induced CCL3 secretion in PBMCs and found that neither *M. tuberculosis*- nor BCG-induced levels of CCL3 differed between patients and healthy controls (Table 3).

### Reduced CCL2 but Increased IFNγ Secretion to *M. tuberculosis* in Patients with Localized ETB Compared to PTB

Previous studies have shown that *Mycobacterium* induced host immune responses differ in pulmonary and extrapulmonary TB [23], [25]. We investigated the association between chemokine and cytokine activation in the tuberculosis patients and the clinical severity of their disease by studying patients with either pulmonary (PTB) or extrapulmonary disease (ETB). Patients were further stratified into either minimal (min-PTB) or moderately advanced (mod-PTB) disease in the pulmonary group on the basis of lung tissue involvement [37] Extrapulmonary tuberculosis patients were stratified accordingly to WHO clinical severity guidelines [47] into less severe localized disease (L-ETB) or severe disseminated disease (D-ETB).

**Figure 1:**
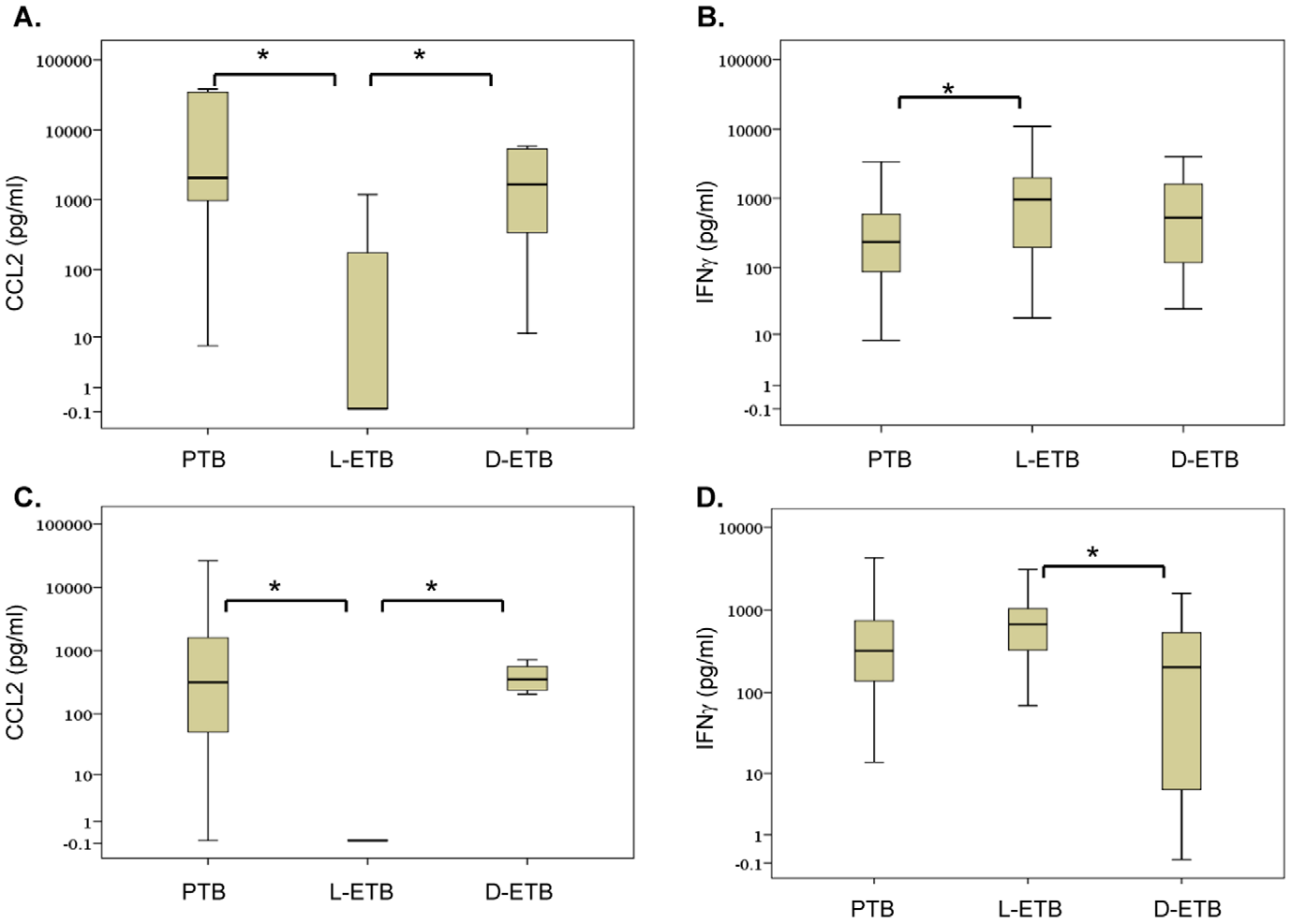
Differential *M. tuberculosis*- and BCG- induced CCL2 and IFNγ responses with TB clinical disease severity. PBMCs (10^6^) were infected with *M. tuberculosis* or BCG (10^6^ CFU) for 18 h after which cell supernatants were harvested for the measurement of cytokines and chemokines. The box plots represent the data for each group after the level of cytokine secretion from unstimulated cells was subtracted. The whiskers indicate the 25th and 75th quartiles, while a line indicating the median separates the two. ‘*’, denotes significant differences between groups (p<0.05) using the Mann-Whitney U test. The data show A) *M. tuberculosis*-induced CCL2 responses of PBMCs from patients with pulmonary tuberculosis (PTB, n = 34) and extrapulmonary TB with less severe localized (L-ETB, n = 16) and severe disseminated (D-ETB, n = 16) disease, B) *M. tuberculosis*-induced IFNγ responses BCG-induced CCL2 responses (C) and IFNγ responses (D) were obtained from PTB, n = 33; L-ETB, n = 16; D-ETB, n = 14.

We found no significant differences between *M. tuberculosis*-induced CCL2 responses from PBMCs of patients with minimal or moderately advanced PTB (median; min-PTB, 31.5; mod-PTB, 1712 pg/ml, p = 0.16), although the trend of CCL2 secretion was greater in mod-PTB patients. The PTB patient groups were combined for comparison with those of ETB group. As shown in Figure 1A, *M. tuberculosis* –induced CCL2 was significantly greater in patients with PTB as compared with L-ETB (p = 0.001). In addition, CCL2 secreted levels from L-ETB patients were also reduced as compared with those with D-ETB (p<0.001).

*M. tuberculosis*-induced IFNγ responses did not differ significantly between patients with min-PTB and mod-PTB (median: minPTB, 298.1; mod-PTB, 233 pg/ml, p = 0.841). *M. tuberculosis*- induced IFNγ secretion in the combined group of PTB patients was significantly lower than responses observed in the L-ETB group (p = 0.04, Fig. 1B). Surprisingly, no difference was observed between IFNγ levels from D-ETB and L-ETB which may be due to the variability in IFNγ responses between donors within each patient group.

### BCG-Induces Reduced CCL2 and IFNγ Secretion from PBMCs of Patients with D-ETB

We also determined BCG-induced chemokine and cytokine responses (Fig. 1 C and D) in PBMCs of patients with pulmonary and extrapulmonary TB, in order to investigate a relationship between cytokine induction and disease severity in the groups. Overall, BCG-induced CCL2 levels from PTB patients were significantly raised as compared with those from patients with L-ETB (p<0.001, Fig. 1C). BCG-induced CCL2 levels of patients with D-ETB were also greater than those observed in the L-ETB group (p<0.001), Fig. 1C.

BCG-induced IFNγ from PBMCs of L-ETB patients on the other hand was significantly greater than those from patients with D-ETB (p = 0.036), Fig. 1D. Within PTB patients, BCG-induced CCL2 did not differ significantly between minimal and moderate disease (median: min-PTB, 0; mod-PTB, 167.4 pg/ml; p = 0.189) and BCG-induced IFNγ also showed the same trend (median: min-PTB, 673; mod-PTB, 597 pg/ml; p = 0.906).

### *M. tuberculosis* – and BCG-Induced IL10 and CCL3 Responses Do Not Differ between PTB and ETB Severity Groups

*M. tuberculosis*-induced IL10 levels were comparable between PTB and ETB groups (Figure S1A) but there was an increasing trend of IL10 in the disseminated ETB group (median: PTB, 56; L-ETB, 134; D-ETB, 282 pg/ml). Within the PTB patients a higher trend in IL10 was noted in mod-PTB as compared with min-PTB group (median: min-PTB, 22.6 pg/ml; mod-PTB, 483 pg/ml, p = 0.078), however this difference was not significant.

*M. tuberculosis-* induced CCL3 concentrations were found to be comparable between the pulmonary and extrapulmonary TB patients studied (Fig. S1B: median; PTB, 1306; L-ETB, 999 D-ETB, 1471 pg/ml, respectively), as well as between PTB patients with either minimal or moderate disease (median; min-PTB, 974; mod-PTB, 1425; p = 0.653).

BCG-induced IL10 levels were comparable between PTB and E-TB groups (Fig. S1C) although there was an increasing trend of IL10 in the disseminated ETB group (median: PTB, 36; L-ETB, 69; D-ETB, 93 pg/ml). Although not significant, again BCG-induced IL10 responses of PBMCs showed a higher trend in mod-PTB as compared with min-PTB group (median: min-PTB, 35.9 pg/ml; mod-PTB, 311 pg/ml, p = 0.228). Association of IL10 with increasing pathology supports the hypothesis that IL10 may play a role in reducing collateral tissue damage [48].

BCG- induced CCL3 was comparable between patients with either minimal or moderate PTB (median; min-PTB, 974; mod-PTB, 1425; p = 0.834). BCG*-* induced CCL3 concentrations were found to be comparable between the pulmonary and extrapulmonary TB patients studied (Fig. S1D; median: PTB, 1592; L-ETB, 1056; D-ETB, 1296 pg/ml, respectively), indicating an absence of association of CCL3 with TB disease severity.

### Differential CCL2 and TNFα Gene Expression in Pulmonary and Extrapulmonary Tuberculosis

**Figure 2:**
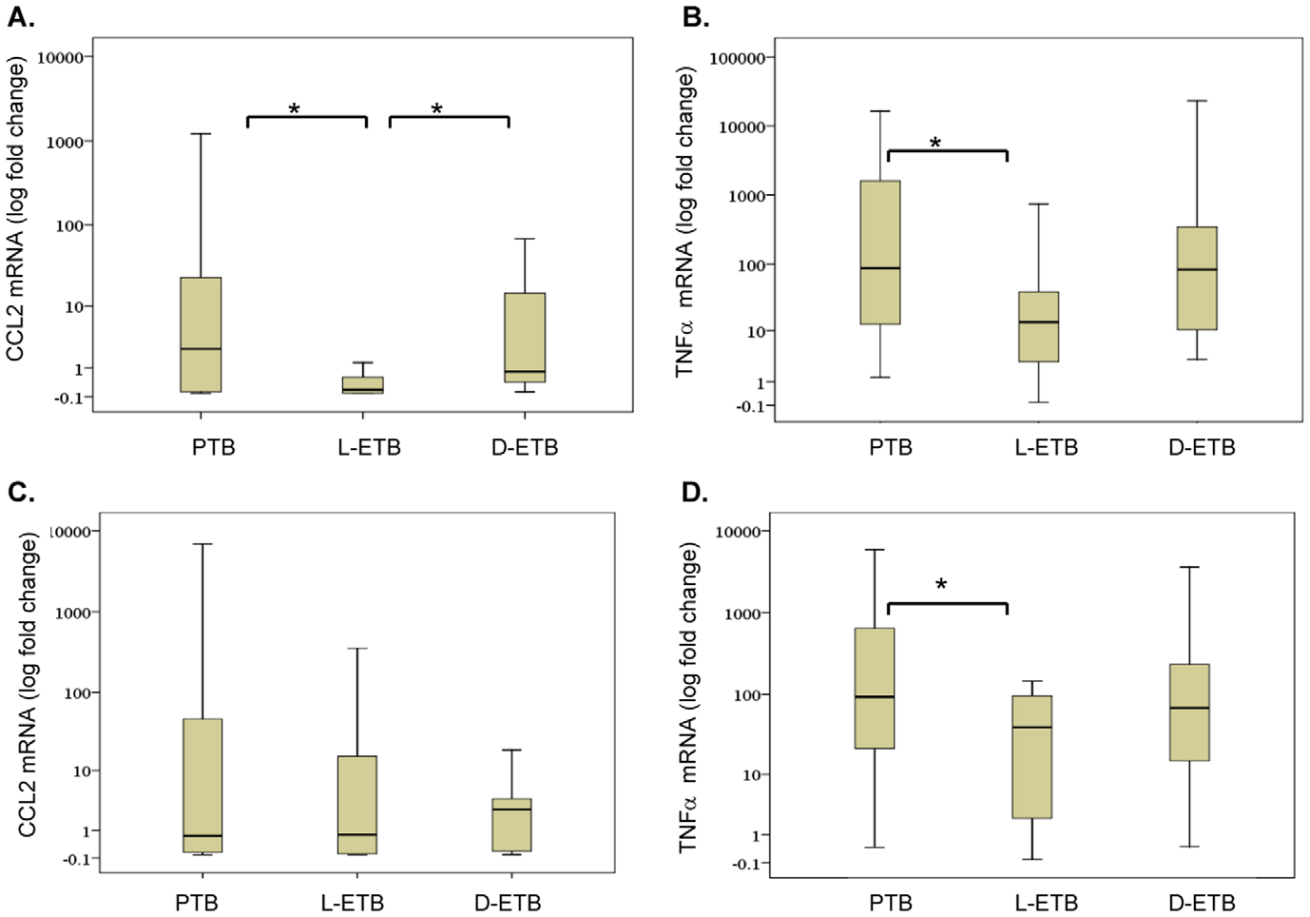
Differential *M. tuberculosis*-induced CCL2 and TNFα mRNA expression in pulmonary and extrapulmonary TB. RNA was extracted from *Mycobacterium*-infected PBMCs after 18 h post stimulation and subjected to RTPCR for chemokine and cytokine genes. Box plots depict fold increase in gene expression after normalization to the housekeeping gene HuPO. The whiskers indicate the 25th and 75th quartiles, while a line indicating the median separates the two. ‘*’, p<0.05, indicate differences between groups using the Mann-Whitney U test. Data is depicted as fold increase in each target gene per 100 copies. *M. tuberculosis* -induced mRNA expression of A) CCL2, and B) TNFα is shown for PTB, n = 22; L-ETB, n = 15, D-ETB, n = 13 patients. BCG-induced mRNA expression of C) CCL2 and D) TNFα is shown for PTB, n = 16; L-ETB, n = 14; L-ETB, n = 14 patients. ‘*’, p<0.05, indicate differences between groups using the Mann-Whitney U test, TNFa (TNFα).

Many reports which have investigated chemokine responses to *M. tuberculosis* in patients have focused on either protein secretion or gene expression responses. To determine whether the secretory patterns we observed matched gene expression trends we investigated *M. tuberculosis* -induced mRNA transcripts in stimulated PBMCs by studying CCL2 and CCL3 expression. As CCL2 and CCL3 expression is regulated by TNFα, a critical activator of macrophages [49], [50] we also determined the expression of TNFα. In addition, we investigated the expression of CXCL8, which is responsible for neutrophil recruitment and has been shown to play a role in tuberculosis infections [51]. We determined gene expression in both pulmonary and extrapulmonary TB groups. *M. tuberculosis* infection of PBMCs resulted in significantly greater CCL2 expression in PBMCs of PTB patients (Fig. 2A) as compared to those with L-ETB (p = 0.023). *M. tuberculosis* induced CCL2 expression was also increased in D-ETB patients as compared with L-ETB (p = 0.005). *M. tuberculosis*-induced TNFα was significantly greater in PTB patients (Fig. 2B) as compared with L-ETB (p = 0.02). TNFα expression was also raised in D-ETB as compared with L-ETB (p = 0.09), although this difference was not significant.

BCG-induced CCL2 mRNA expression was lower in PTB as compared with that induced by *M. tuberculosis*. No difference was found between BCG-induced CCL2 mRNA expression between TB groups (Fig. 2A). BCG-induced TNFα was greater in patients with PTB than those with L-ETB (p = 0.03), Fig. 2B.

*M. tuberculosis*- induced CCL3 and CXCL8 mRNA transcripts were comparable between PTB, L-ETB and D-ETB groups, Figure S2 A-B. BCG-induced CCL3 (Fig. S2C) and, –CXCL8 mRNA expression (Fig. S2D) were also comparable between TB groups.

## Discussion

Our data illustrates differences in the activation of immune regulatory chemokines and cytokines in tuberculosis disease with differing site and severity. CCL2 has potent chemotactic and activating properties for monocytes, macrophages, dendritic cells and CD4+ T cells. The most significant finding was that CCL2 was consistently associated with severe disease. We propose that CCL2 could be a useful adjunct marker of severity in tuberculosis.

To evaluate differences between background immune responses due to BCG vaccination and environmental mycobacteria and infection with *M. tuberculosis*, we investigated host immune responses *in vitro* infection with virulent *M. tuberculosis* H37Rv and avirulent *M. bovis* BCG vaccine strains. Both *M. tuberculosis* and BCG elicited an increase in CCL2 in TB patients as compared with controls. CCL2 is responsible for the recruitment of leucocytes to the site of infection and therefore raised CCL2 may be characteristic of granuloma formation and the influx of monocyte driven responses.

Our finding that *M. tuberculosis* infection results in reduced IFNγ secretion in patients as compared with TST+ ECs donors is consistent with previous reports [52]. The BCG-induced IFNγ responses which were depressed in TB patients compared with both TST- ECS and TST+ ECS healthy controls also confirm our previous reports on pulmonary TB patients [53]. All of our ECs were BCG vaccinated so the increased IFNγ responses to BCG may be related to the presence of T memory recall responses in these individuals. The absence of similar T memory recall responses in TB patients could be due to raised IL10 in TB patients as compared with healthy controls, resulting in down modulation of T cell responses in TB patients [54].

CCL3 is a macrophage and T cell attractant, activated by *M. tuberculosis* infection of host cells [55], [56]. We found *M. tuberculosis* and BCG both induced CCL3 secretion and gene expression in PBMCs. However, we found no association of CCL3 (either protein levels or gene expression) between tuberculosis patients and healthy controls in response to *M. tuberculosis* or BCG stimulation.

While a number of studies have utilized both microarray and RT-PCR studies to analyze *Mycobacterium*-induced expression in macrophages, most of these studies have been performed in murine cells [57], [58]. A study by Ragno *et* al. showed *M. tuberculosis* induced changes in the THP-1 monocytic cell line, where it was reported that a number of chemokines such as CCL2, CCL3, CXCL8 in addition to cell surface adhesion molecules ICAM and integrins were upregulated post-infection [59]. There is limited data on gene expression profiles in TB patients with differing severity of disease.

While the value of BCG vaccination in early childhood to prevent disseminated disease is widely accepted the value of BCG vaccination in adult population particularly for pulmonary disease has been challenged in high burden countries [60]. The number of patients who were BCG vaccinated in the patient groups varied; with a greater proportion in L-ETB (82%) than those with D-ETB (40%). There is very limited data on the protection provided by BCG in adult tuberculosis and although this data is too small to draw any conclusions it suggests that BCG vaccination may result in a preponderance of less severe extrapulmonary TB disease.

The data available regarding age of ETB patients is variable according to region and ethnicity of the study populations, with ETB associated with younger age (<25) and females in African and Asian patients [61]–[63]. Reports from Turkey show that the predominant age range studies is 25–44 y for ETB patients [2]. Our TB patients were of the range 24.5–31.5 y, and within this we found ETB patients to be older than those with PTB (p = 0.034). This may also not be a contradictory result as all of our patients were already in a younger age range. A larger number of samples with a broader age range need to be analyzed for further confirmation.

When responses between patients with pulmonary and extrapulmonary TB were compared, *M. tuberculosis* and BCG-induced CCL2 secretion was found to be increased in patients with pulmonary TB as compared with those with less severe localized extrapulmonary TB. *M. tuberculosis-* induced secretion and mRNA expression of CCL2 was greater in PTB than in L-ETB, and also reduced in L-ETB as compared with D-ETB patients. Our comparison of *M. tuberculosis* and BCG-induced responses in patients illustrated that although the trend of response to the mycobacteria was similar, the magnitude of responses to the virulent mycobacteria was greater than that to attenuated BCG. As the L-ETB group consisted of patients with tuberculous lymphadenopathy, this may indicate more active monocyte and T cell recruitment in disease localized to the lung parenchyma as compared to lymph nodes. It also supports previous reports of increased CCL2 secretion in cells of TB patients with pulmonary disease [23], [64].

The raised CCL2 responses in patients with severe disseminated D-ETB (spinal, tuberculous meningitis and abdominal TB) as compared with L-ETB, also indicate that increased CCL2 is associated with increasing disease severity. This fits with previous work which has shown that levels of inflammatory chemokines are increased in body fluids of patients with extrapulmonary disseminated infections TB such as in tuberculous meningitis, spinal tuberculosis or miliary disease [65]–[67].

Raised *M. tuberculosis* - induced IFNγ in localized L-ETB as compared with pulmonary TB corresponds with previous studies employing mycobacterial antigen ESAT6 driven responses [25]. The WHO clinical classification of tuberculosis disease severity lists tuberculous lymphadenitis as the least severe form of TB. The increased effector T cell IFNγ response in the L-ETB group as compared with pulmonary TB may reflect a reduction in IFNγ with increasing mycobacterial load, inflammation and clinical severity. We have also previously reported an inverse relationship of IFNγ levels in response to *M. tuberculosis* culture filtrate proteins with clinical severity in both PTB [68] and ETB [69]. BCG-induced IFNγ was raised in L-ETB but only when compared with the D-ETB group. As there were more BCG vaccinees in the L-ETB group this may reflect increased *M. tuberculosis*-specific T cell responses than in the D-ETB group. This is consistent with the role of IFNγ as a potent activator of macrophages for mycobacterial killing and stasis [70].

We did not observe any difference in *M. tuberculosis* induced CCL3 secretion, or CCL3 mRNA expression between patients with pulmonary or extrapulmonary TB with disease in single or multiple sites. Reports by Qiu *et* al. have shown CCL3, CXCL10 and their receptors CCR3, CCR4 and CXCR3 to be upregulated in an unbalanced manner in severe TB in the macaque model [71]. However, in the same study they observed low antigen specific cellular responses in the severely infected macaques [72], indicating a reduced ability of the immune cells to respond to a subsequent challenge with *M. tuberculosis*. Previously, increasing levels of CXCL8 have been shown to be associated with fatal tuberculosis [73]. Therefore, the lack of difference in CXCL8 transcription observed in localized and severe ETB may be due to an antigen specific anergy in severe disease.

TNFα has previously been associated with increasing bacterial load and to be responsible for disease progression in unregulated granuloma formation [74]. Our data showing an increase in CCL2 and TNFα in response to *M. tuberculosis* infection agrees with previous work in murine bone marrow derived macrophages by Kahnert *et* al. [75].

The differences in chemokine and cytokine responses of TB patients elicited by *M. tuberculosis* and BCG indicate that *M. tuberculosis*-specific immune responses remain detectable even in highly endemic populations, where there may be high background responses to environmental mycobacteria. However, such background variability is reflected in the highly variable responses observed in all patient groups as well as in the control groups. Tuberculosis represents an immune spectrum across clinical and subclinical (latent) infection which can only be defined by the host immune response [76], [77]. Clinical studies in humans are therefore limited due to the highly polymorphic and multifactorial nature of the immune responses in a background of variable exposure to cross reactive stimuli.

Overall, these data shows that using CCL2 could provide an adjunct marker of disease severity. In less severe ETB we found the highest IFNγ responses, but lowest CCL2, TNFα and IL10 responses. The coordinate increase in CCL2 and TNFα responses observed in the pulmonary TB group, may indicate active monocyte recruitment to the lungs which are likely to facilitate granuloma formation and localization of *M. tuberculosis* infection. Only CCL2 was increased in severe ETB. Therefore, this suggests that without supportive TNFα regulation CCL2 driven leucocyte activation may not be effective. As a consequence, raised CCL2 in itself may be associated with clinical disease severity and dissemination of infection in the host. It is possible that CCL2 may have a better predictive power when combined with other yet unidentified markers. Larger scale studies are required to further define the role of CCL2 in clinical tuberculosis.

## Supporting Information

Figure S1 *M. tuberculosis*- and BCG- induced IL10 and CCL3 responses in TB patients. PBMCs (10^6^) were infected with *M. tuberculosis* or BCG (10^6^ CFU) for 18 h after which cell supernatants were harvested for the measurement of cytokines and chemokines. The box plots represent the data for each group after the level of cytokine secretion from unstimulated cells was subtracted. The whiskers indicate the 25th and 75th quartiles, while a line indicating the median separates the two. ‘*’ denotes significant differences between groups (p<0.05) using the Mann-Whitney U test. The data show A) *M. tuberculosis*-induced IL10 (A) and CCL3 (B) responses of PBMCs from patients with pulmonary tuberculosis (PTB, n = 34) and extrapulmonary TB with limited (L-ETB, n = 16) and disseminated (D-ETB, n = 16) disease. BCG-induced IL10 (C) and CCL3 responses (D) were obtained from PTB, n = 33; L-ETB, n = 16; D-ETB, n = 14.
(5.09 MB TIF)

Figure S2 *M. tuberculosis*- and BCG-induced CCL3 and CXCL8 mRNA expression in pulmonary and extrapulmonary TB patients. RNA was extracted from *M. tuberculosis*- or BCG-infected PBMCs after 18 h post stimulation and subjected to RTPCR for chemokine and cytokine genes. Graphs depict fold increase in gene expression after normalization to the housekeeping gene HuPO. Data is depicted as fold increase in each target gene per 100 copies. Box plots depict fold increase in gene expression after normalization to the housekeeping gene HuPO. The whiskers indicate the 25th and 75th quartiles, while a line indicating the median separates the two. ‘*’, p<0.05, indicate differences between groups. *M. tuberculosis* -induced mRNA expression of A) CCL3, and B) CXCL8 is shown for PTB, n = 22; L-ETB, n = 15, D-ETB, n = 13 patients. BCG-induced mRNA expression of C) CCL3 and D) CXCL8 is shown for PTB, n = 16; L-ETB, n = 14; L-ETB, n = 14 patients.
(4.85 MB TIF)
