# Supplementary figures and images for: CCL2 Responses to Mycobacterium tuberculosis Are Associated with Disease Severity in Tuberculosis
Source: PLoS One. 2009 Dec 29;4(12):e8459. doi: 10.1371/journal.pone.0008459 (PMC2793516; doi:10.1371/journal.pone.0008459)

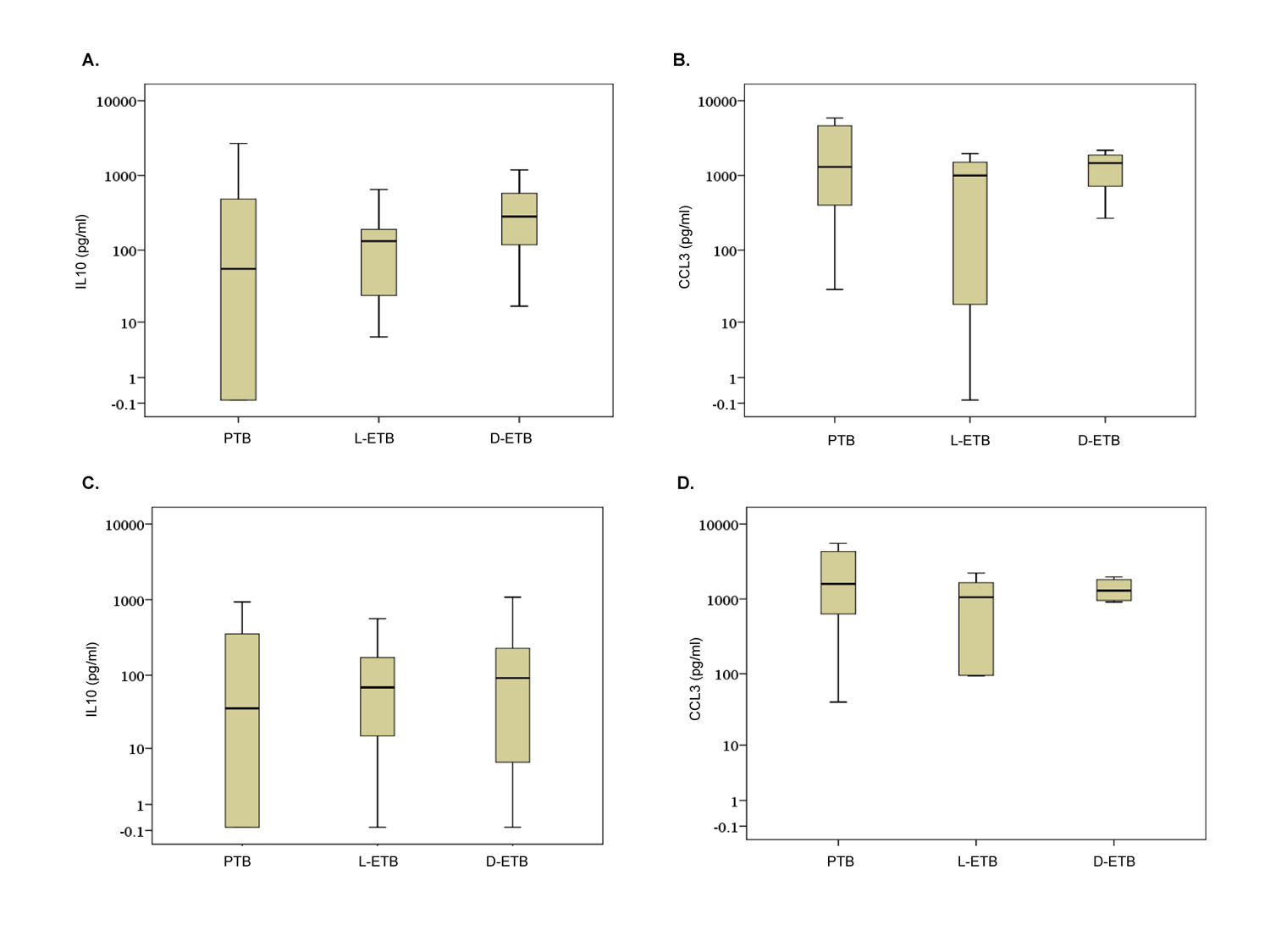

Supplement: Figure S1 — M. tuberculosis- and BCG- induced IL10 and CCL3 responses in TB patients. PBMCs (106) were infected with M. tuberculosis or BCG (106 CFU) for 18 h after which cell supernatants were harvested for the measurement of cytokines and chemokines. The box plots represent the data for each group after the level of cytokine secretion from unstimulated cells was subtracted. The whiskers indicate the 25th and 75th quartiles, while a line indicating the median separates the two. ‘*’ denotes significant differences between groups (p<0.05) using the Mann-Whitney U test. The data show A) M. tuberculosis-induced IL10 (A) and CCL3 (B) responses of PBMCs from patients with pulmonary tuberculosis (PTB, n = 34) and extrapulmonary TB with limited (L-ETB, n = 16) and disseminated (D-ETB, n = 16) disease. BCG-induced IL10 (C) and CCL3 responses (D) were obtained from PTB, n = 33; L-ETB, n = 16; D-ETB, n = 14. (5.09 MB TIF) [file pone.0008459.s001.tif]

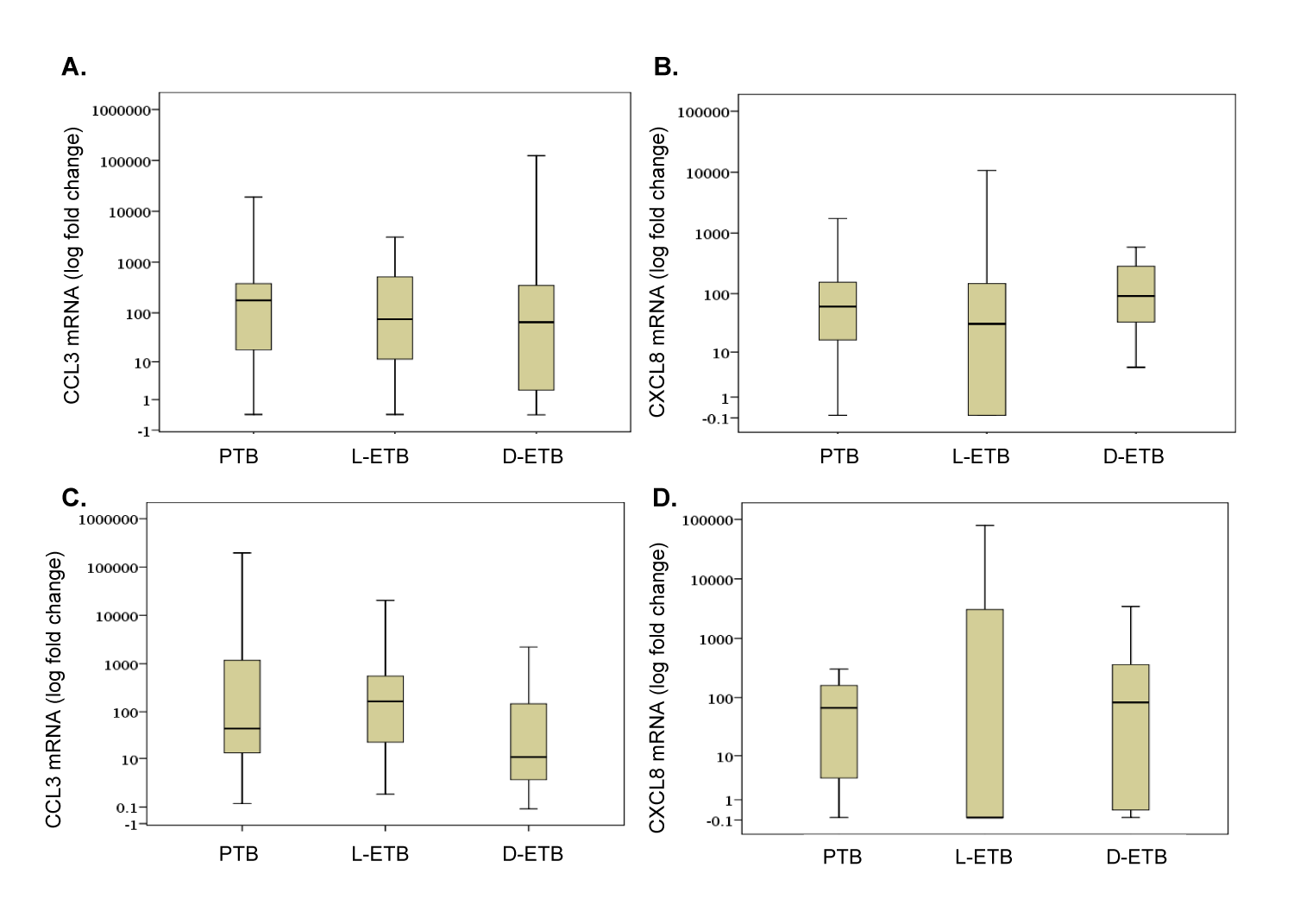

Supplement: Figure S2 — M. tuberculosis- and BCG-induced CCL3 and CXCL8 mRNA expression in pulmonary and extrapulmonary TB patients. RNA was extracted from M. tuberculosis- or BCG-infected PBMCs after 18 h post stimulation and subjected to RTPCR for chemokine and cytokine genes. Graphs depict fold increase in gene expression after normalization to the housekeeping gene HuPO. Data is depicted as fold increase in each target gene per 100 copies. Box plots depict fold increase in gene expression after normalization to the housekeeping gene HuPO. The whiskers indicate the 25th and 75th quartiles, while a line indicating the median separates the two. ‘*’, p<0.05, indicate differences between groups. M. tuberculosis -induced mRNA expression of A) CCL3, and B) CXCL8 is shown for PTB, n = 22; L-ETB, n = 15, D-ETB, n = 13 patients. BCG-induced mRNA expression of C) CCL3 and D) CXCL8 is shown for PTB, n = 16; L-ETB, n = 14; L-ETB, n = 14 patients. (4.85 MB TIF) [file pone.0008459.s002.tif]
